# Supplementary material for: Maternal perceptions of father involvement among refugee and disadvantaged families in Beirut, Lebanon
Source: PLoS One. 2020 Mar 5;15(3):e0229670. doi: 10.1371/journal.pone.0229670 (PMC7058288; doi:10.1371/journal.pone.0229670)
Supplement: S1 Table — (PDF) [file pone.0229670.s001.pdf]

S1 Table. Sample Demographic Characteristics.

Characteristic	Min	Max	*M*	*SD*	*n*	%
Child participating in MOCEP						
Age (years)	2.05	7.93	4.34	1.24		
Gender (male)					54	51.9
Mother						
Age (years)	20	53	32.36	5.82		
Lived in current community (years)	0.25	53	19.56	14.26		
Place of birth						
Lebanon					71	68.3%
Another country					33	31.7%
Marital status						
Married					103	99.0
Divorced					1	1.0
Cultural heritage						
Palestinian					46	44.2
Lebanese					39	37.5
Syrian					18	17.3
Other					1	1.0
Highest level of education						
Primary school					16	15.4
Elementary school					52	50.0
High school					17	16.3
Technical school					8	7.7
University (BA, License)					11	10.6
Employed (yes)					11	10.6
Father						
Relationship to child						
Father					103	99.0
Stepfather					1	1.0
Father lives with mother and child (yes)					94	90.4
Cultural heritage						
Palestinian					58	55.8
Lebanese					36	34.6
Syrian					10	9.6
Highest level of education						
No school (illiterate)					1	1.0
Primary school					20	19.2
Elementary school					54	51.9
High school					10	9.6
Technical school					6	5.8
University (BA, License)					10	9.6
Missing					3	2.9
Employed (yes)					88	84.6
Missing					2	1.9
