# Supplementary material for: Maternal perceptions of father involvement among refugee and disadvantaged families in Beirut, Lebanon
Source: PLoS One. 2020 Mar 5;15(3):e0229670. doi: 10.1371/journal.pone.0229670 (PMC7058288; doi:10.1371/journal.pone.0229670)
Supplement: S2 Table — (PDF) [file pone.0229670.s002.pdf]

S2 Table. Father Involvement Questionnaire (FIQ).

My husband…	*Subscale*
A. Is knowledgeable about our child(ren)'s education	C
B. Listens to our child(ren)	C
C. Speaks often with our child(dren)	C
D. Is not too harsh when disciplining our child(ren)	C
E. Plays with our child(ren)	C
F. Reads with, or to, our child(ren)	C
G. Enjoys spending time with our child(ren)	C
H. Often yells when disciplining our child(ren) (R)	C
I. Hits when disciplining our child(ren) (R)	C
J. Is tolerant with me	M
K. Treats me well in front of our child(ren)	M
L. Usually speaks to me in a respectful manner	M
M. Shares his views about our child(ren) with me	M
N. Listens to my opinion about raising our child(ren)	M
O. Supports me when I discipline my child(ren)	M
P. Attends community events often	Co
Q. Usually attends community events on his own	Co
R. Usually attends community events with family members	Co
S. Spends time with other fathers in the community	Co
T. Enjoys spending time with other families	Co

C = Father-Child Interaction; M = Father-Mother Interaction; Co = Father-Community Interaction. (R) = reverse coded item. The instructions are: "*For this set of questions, please think about the way your husband interacts with your child(ren), with you and with other around him".* All items are rated on the following 4-point scale: 1 = *Strongly disagree*, 2 = *Disagree*, 3 = *Agree*, 4 = *Strongly agree*.
