# Supplementary material for: Maternal perceptions of father involvement among refugee and disadvantaged families in Beirut, Lebanon
Source: PLoS One. 2020 Mar 5;15(3):e0229670. doi: 10.1371/journal.pone.0229670 (PMC7058288; doi:10.1371/journal.pone.0229670)
Supplement: Attachment — Submitted filename: Reponse to reviewers.docx [file pone.0229670.s004.docx]

Reviewer #1: The authors did an excellent job addressing prior comments and concerns. I only noticed a few typos that I wanted to point out:

- p. 23, para. 1: change "infant" to "infancy"
- p. 23, para. 1: change "as well as" to "and" in the sentence that starts with "Importantly, maternal distress..."
- p. 24, para. 2: change "comprised of" to "composed of"

**Response:** Thank you for pointing out these grammatical issues. All issues have been corrected.

Reviewer #2: The authors have done a very good job in attending to comments and suggestions previously offered, leading to further strengthening of a manuscript that was already very strong.

I have just two substantive comments to offer as they finalize this work for publication.

The first is that the authors temper the conclusions they offer about what they see as the lack of associations involving mothers’ social support, for a couple of reasons.

**Comment 1:** First, the fact that a particular path coefficients does not meet the cutoff of p < .05 does not mean that the construct is actually of no importance in this sample (or elsewhere in the real world). Absolute values of coefficients that are interpreted as significant in this study range from as low as .19 to as high as .45. The authors have not conducted significance testing to determine, for example, that coefficients for dotted paths described as non-significant are in fact statistically significant from those coefficients that they have reported as significant (and thus, by implication, of “real world importance”). Thus, I believe it is an over interpretation to conclude, in this statement, that ”paternal involvement (but not perceived social support) may alleviate the stress the mother experiences in being a parent in under-resourced or at-risk circumstances.”

Personally, I would suggest just leaving out the one sentence above, that is currently between the following two, and instead simply state that “These findings support the notion that both constructs may function as distinct sources to promote maternal well-being...... Together, these findings support the notion that maternal distress may mediate the association between maternal perceptions of paternal involvement and both maternal parenting stress and maternal harsh discipline.”

**Response:** We appreciate this comment. Both constructs were indeed associated with maternal well-being. Perceived social support was associated with maternal distress as well, despite to a lesser degree (beta = -0.095) compared to maternal well-being (beta = 0.194). Thus, we agree to take out the sentence as suggested by the reviewer.

**Comment 2:** Another issue to consider is that these authors’ findings on social support may have rested partly on measurement issues. It seems that at least 3 of the 7 items on support had to do with frequency of connecting with close others. For a mother with an uninvolved, unsupportive husband, who is sole caregiver of her children, there would be obvious constraints in how much she can visit with others in her daily life.

I would like to emphasize that this is an issue of importance not just for science but as importantly, for what is conveyed to lay folk and policy makers. In other words, I would urge the authors to consider carefully the message that they are disseminating regarding what social supports can, and *cannot* do, for mothers at risk. Indeed, the NASEM (2019) consensus report cited has conclusively stated that the presence of dependable supports is actually the single most important protective factor for all types of moms at risk (and therefore, for their children). For the many single or unhappily married mothers in the world, it would be disheartening indeed to hear the message, from these authoritative authors, that if the child’s father remains uninvolved, other caring relationships in their lives will be of no use to them in terms of bolstering their well-being and parenting.

If the authors do in fact wish to retain this suggestion, then I would very much like to see a conceptual explanation of why their conclusions depart so drastically from those unequivocally stated in the NASEM review.

**Response:** We carefully reviewed the manuscript (particularly the Discussion section) to make sure we do not (unintentionally) minimize the role of social support in the lives of mothers at risk. As evidenced by the bivariate correlations, perceived social support was positively associated with maternal well-being (*r* = .27) and negatively associated with maternal distress (*r* = -.28).

We added the following text to emphasize the important role of social support: “The findings of our study support this notion. As evidenced by the bivariate correlations, higher levels of perceived social support were associated with higher levels of subjective maternal well-being and lower ratings of maternal distress. Thus, although our focus was on father-mother relations, we fully acknowledge that supportive relations manifest among wider networks.“

**Comment 3:** The second issue concerns these statements on P 31:
“Anecdotal comments from fathers in refugee settings revealed various unique concerns and worries that may prevent fathers from being maximally involved with their family. Common stressors include a feeling of powerlessness due to underemployment and lack of ability to provide for one’s family. Perceived isolation and discrimination from the larger community may also highly affect the fathers’ well-being. Together, these extreme stressors may affect fathers’ well-being more generally, which can compromise their levels of involvement. These anecdotal observations may reveal key issues that need to be targeted in any program. “

I see these comments as in fact providing rich insights into why it is hard for fathers to be more involved with their families. At the same time, I’m left disappointed with the directions offered for future interventions. In other words, would a new family-based program actually reduce the major listed concerns for fathers, such as “underemployment and lack of ability to provide for one’s family (and) perceived isolation and discrimination“? If the authors have suggestions of strategies for how these could realistically be done, it would be wonderful to hear these spelled out. If they do not, it would be useful to acknowledge that these are clearly critical issues, but not easily solved at the level of family-based interventions - they will need changes at larger levels of national policies and perhaps, community norms.

In sum, I congratulate these authors again for a very fine piece of work! I have no doubt that it will make a strong contribution to the literature.

**Response:** Thank you for your thoughtful comment. We agree that strong implications for policy and programming are a desirable outcome of any research. However, we are careful not to overstate the informative value of our data.

We added the following text to convey our recommendations for future studies aimed at developing policy/program recommendations: “These are critical issues from the perceptive of the fathers we interviewed. However, they are neither solely nor easily solved at the level of family-based intervention. Among many factors, cultural, gender, and migration dynamics, as well as community norms and national policies need to be considered to devise strong policy and/or program recommendations, which are outside the scope of this work. The incremental contribution of this study is to substantiate the role of paternal engagement and support, with further research needed to develop effective programs and strategies that may reduce the major concerns voiced by fathers.”
